# Design and Evaluation of New Gel-Based Floating Matrix Tablets Utilizing the Sublimation Technique for Gastroretentive Drug Delivery

**DOI:** 10.3390/gels10090581

**Published:** 2024-09-09

**Authors:** Worawut Kriangkrai, Satit Puttipipatkhachorn, Pornsak Sriamornsak, Srisagul Sungthongjeen

**Affiliations:** 1 Department of Pharmaceutical Technology, Faculty of Pharmaceutical Sciences and Center of Excellence for Innovation in Chemistry, Naresuan University, Phitsanulok 65000, Thailand; wg.kriangkrai@gmail.com; 2 Center of Excellence for Natural Health Product Innovation, Naresuan University, Phitsanulok 65000, Thailand; 3 Department of Manufacturing Pharmacy, Faculty of Pharmacy, Mahidol University, Bangkok 10400, Thailand; satit.put@mahidol.ac.th; 4 Department of Industrial Pharmacy, Faculty of Pharmacy, Silpakorn University, Nakhon Pathom 73000, Thailand; sriamornsak_p@su.ac.th

**Keywords:** floating tablets, sublimation technique, gastroretentive drug delivery, hydroxypropyl methylcellulose, drug release kinetics

## Abstract

A gel-based floating matrix tablet was formulated and evaluated using the sublimation technique to enhance gastroretentive drug delivery. Anhydrous theophylline was employed as the active pharmaceutical ingredient, combined with sublimation agents and hydroxypropyl methylcellulose as the gel-forming polymer. The resulting tablets exhibited high porosity, immediate floatation, and sustained buoyancy for over 8 h. Optimization of the floating behavior and drug release profiles was achieved by adjusting the viscosity of and hydroxypropyl methylcellulose and the concentration of sublimation agents, specifically ammonium carbonate and menthol. These agents were selected for their effectiveness in creating a porous structure, thus reducing tablet density and enhancing floatation. Higher HPMC viscosity resulted in increased floating force, slower drug release, and improved swelling properties due to a slower erosion rate. A critical assessment of the balance between tablet porosity, mechanical strength, and drug release kinetics indicates that ammonium carbonate provided superior tablet hardness and lower friability compared to menthol, favoring a controlled release mechanism. The release dynamics of theophylline were best described by the anomalous (non-Fickian) diffusion model, suggesting a combined effect of diffusion and erosion. This research advances the development of gastroretentive drug delivery systems, highlighting the potential of sublimation-based floating matrix tablets for sustained drug release.

## 1. Introduction

Floating drug delivery systems (FDDSs) have been developed to achieve prolonged gastric retention times and enhance drug bioavailabilitiess [1,2,3]. These systems benefit drugs with an absorption window in the stomach or upper small intestine, such as furosemide [4,5] and norfloxacin [6], and those poorly soluble or unstable in intestinal fluid, such as captopril [7], diazepam [2,8], and verapamil HCl [9]. Additionally, FDDSs are suitable for locally acting drugs in the proximal gastrointestinal tract, such as antibiotics for *Helicobacter pylori* eradication in peptic ulcer treatment [10]. FDDSs can be classified into noneffervescent and effervescent systems. Effervescent systems generate gas via the neutralization of sodium bicarbonate by gastric acidity, enabling floatation [11]. However, the floatation time can be unpredictable due to variable gastric pH and contents [12], and the basic microenvironment created by sodium bicarbonate might interact with and degrade active drugs. Noneffervescent FDDSs, such as hollow microspheres, granules, and tablets, immediately float and are less affected by gastric pH differences. Techniques like solvent evaporation create floating microballoons with spherical cavities in the drug-loaded polymer shell, with floating properties and drug release profiles depending on the polymer, plasticizer, and solvent used [13,14,15,16].

Streubel et al. [9,17] developed gastroretentive systems using low-density foam powder, specifically polypropylene foam powder, to prolong the gastric retention time. Low-density porous carriers with a high surface area [18] and materials like edible oils have also been explored to decrease the density of dosage forms [4]. Among noneffervescent FDDS techniques, the sublimation technique has gained popularity. Fukuda et al. [12] prepared floating tablets by sublimating a menthol core at elevated temperatures, creating hollow tablets that float immediately and remain buoyant regardless of gastric pH fluctuations. Despite its potential, the sublimation technique for FDDSs has been underexplored [19,20,21].

Hence, the objective of the present work was to develop the floating matrix tablet with an intrinsic density lower than that of gastric fluid (1.004 g/cm^3^) and sustained drug release using sublimation substances. Further, the effects of various formulation variables on the floating properties and drug release profiles of the floating tablet were investigated to achieve the desired system properties.

## 2. Results and Discussion

### 2.1. Design of the Floating Matrix Tablets

**Figure 1:**
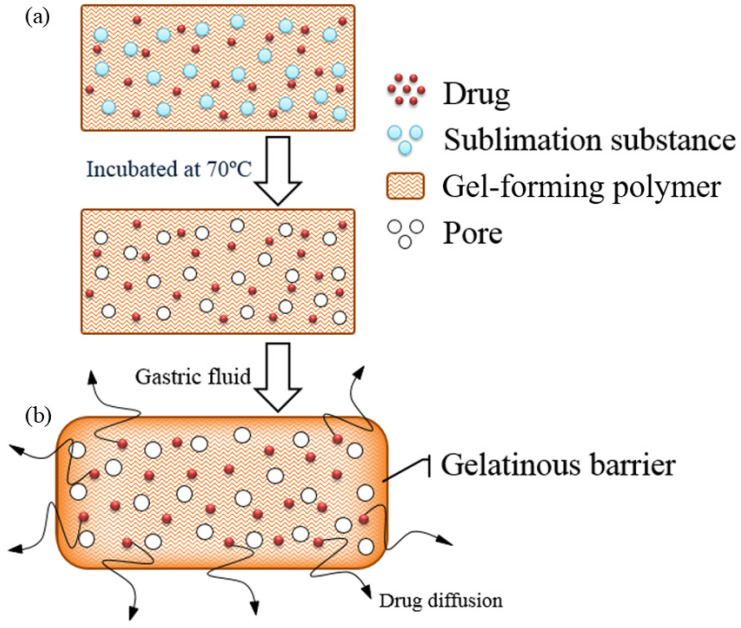
Schematic of the floating matrix tablet prepared by the sublimation technique (**a**). The gelatinous barrier of the hydrated hydroxypropyl methylcellulose (HPMC) leads to air entrapment and retards the drug release (**b**).

The floating matrix tablets were composed of drug (theophylline), sublimation substances, and gel-forming polymer (Figure 1a). The mixed excipients were compressed into tablets and incubated in an oven (70 °C) for 72 h. During incubation, the sublimation substance evaporated creating a porous structure that reduced the tablet density and allowed for buoyancy. Figure 1b shows the floating mechanism of the tablets prepared using the sublimation technique. Because of this process, tablets with a low density (<1 g/cm^3^) immediately floated upon contact with the acidic medium (0.1 N HCl). The gel-forming polymer swelled and formed a viscous gel layer, retaining the tablet’s bulk density and shape integrity. This outer gelatinous barrier also acted as a hydrated boundary membrane, controlling drug release through slow diffusion.

**Figure 2:**
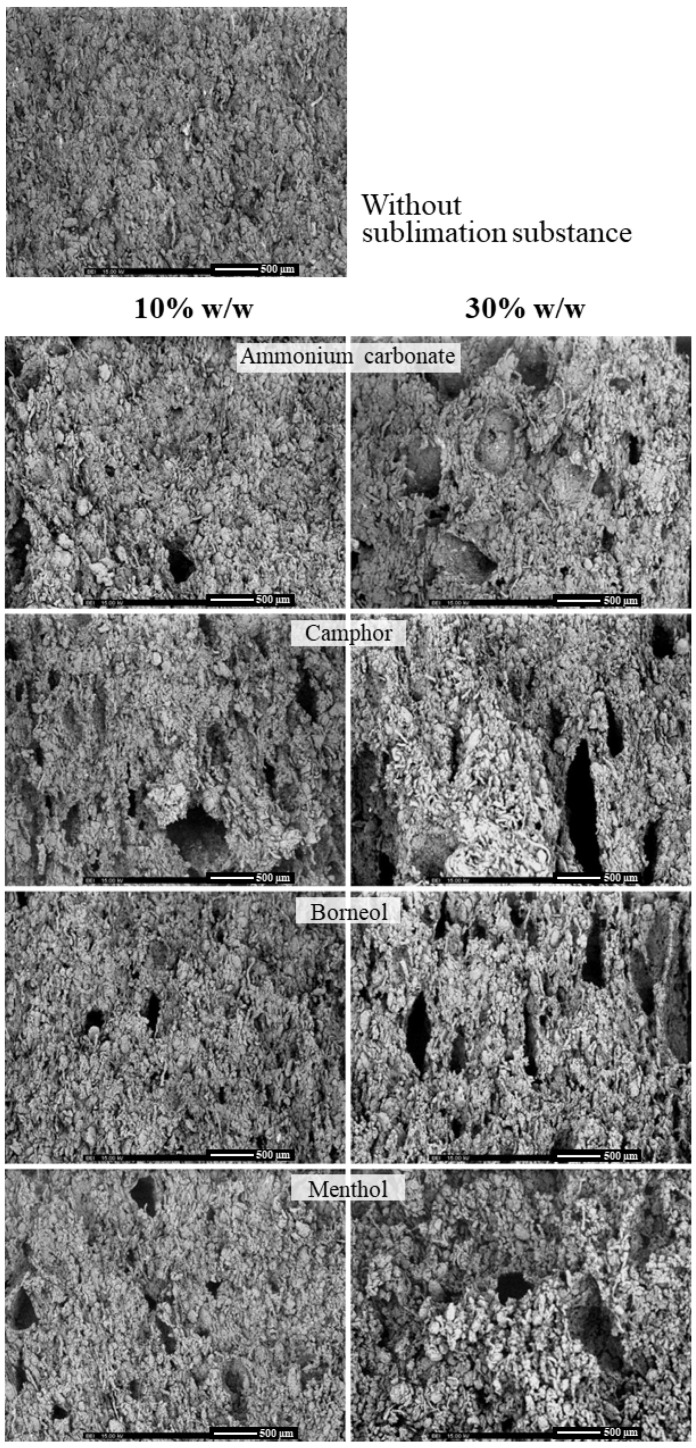
SEM of cross-sections of the floating tablets after sublimation at 30× magnification.

The cross-sectional morphology of the floating matrix tablets observed via SEM is shown in Figure 2. The tablet without the sublimation substance exhibited a dense and homogenous structure, whereas those prepared with the sublimation technique clearly displayed a porous structure. These results correlate well with the further density data of the tablets. The characteristics of the porous structure depended on the type and amount of the sublimation substance used. Tablets with ammonium carbonate exhibited the smallest pore size, indicating the highest compressibility. Increased amounts of sublimation substance led to greater porosity, with more pores present in the tablets.

### 2.2. Physical Properties of the Floating Matrix Tablets

Density is a crucial parameter for predicting the floatability of a floating dosage form [17,19]. The influence of different sublimation substances (ammonium carbonate, camphor, borneol, and menthol) and their quantities on tablet properties were investigated and summarized in Table 1.

Incorporating sublimation substances into the matrix tablets provided a density range of 0.46–1.06 g/cm^3^, depending on the type and amount used. During heat treatment, the tablets lost weight through the sublimation process, creating a porous structure that noticeably reduced the tablet density. When using the same amount of each sublimation substance, menthol resulted in the lowest tablet density, followed by camphor, borneol, and ammonium carbonate, respectively.

The drug content in the floating matrix tablets remained stable after the preparation process within the range of 96.53–102.24%.

This variation is probably due to the differing compressibilities of each sublimation substance. However, the tablets with menthol demonstrated poor physical properties, such as low hardness and high friability. This was because menthol first liquefied at the sublimation temperature of 70 °C before evaporating to the gas phase. Fukuda and Goto previously reported that molten menthol acts like water, causing tablets with hydroxypropyl methylcellulose (HPMC) to swell and crumble [19]. The high porosity of the tablets correlates directly with the increased levels of sublimation substance, resulting in significant decreases in density and hardness. Previous studies similarly found that sublimating camphor in matrix tablets significantly increases porosity [20,22]. Additionally, the type of HPMC used impacts tablet hardness. Tablets with HPMC K type were found to be 2–3 times harder than those with HPMC E type, suggesting that HPMC K type provides higher compressibility.

### 2.3. Effect of Formulation Variables on Floating Properties and Drug Release of the Floating Matrix Tablets

#### 2.3.1. Effect of Type of Gel-Forming Polymer

A functional floating system should float immediately upon contact with gastric fluid and remain buoyant long enough to prevent transit into the small intestine. Tablets without sublimation substances could not float (Table 2). However, highly porous tablets with an optimal amount of sublimation substance floated immediately because of their low-density porous structure. Upon contact with the medium, the gelatinous barrier maintained the tablets’ shape and buoyancy for 8 h. These findings are in good agreement with those of Oh et al. [20], who reported that camphor-containing tablets with a density of 0.88–0.98 g/cm^3^ exhibited immediate and sustained buoyancy for 24 h.

**Figure 3:**
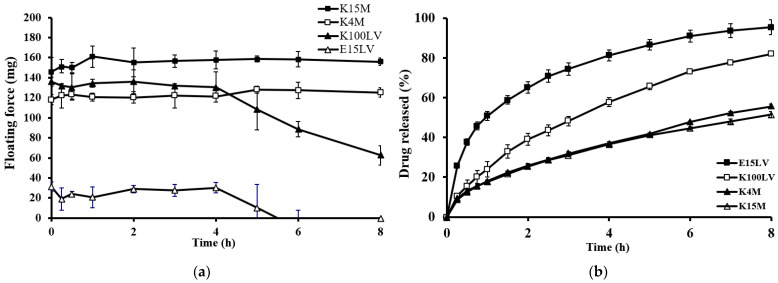
Effect of the grade of HPMC on the floating force (**a**) and theophylline released (**b**) from floating matrix tablets (30% *w*/*w* menthol) in 0.1 N HCl.

Figure 3a presents the floating force–time plot for tablets with different HPMC grades (E15LV, K100LV, K4M, and K15M), each containing 30% *w*/*w* menthol. Tablets with HPMC K15M and K4M notably demonstrated high and constant floating forces due to the high viscosity of the gel layer. In contrast, the tablets with a lower viscosity HPMC (K100LV and E15LV) maintained a constant floating force for approximately 4 h, which then declined as a function of time. The floating force of the tablets with HPMC E15LV was significantly lower than those with HPMC K types. This was due to differences in the proportions of hydroxypropyl and methoxyl substitutions [23]. The rate of hydration increased with the hydrophilic hydroxypropyl group, leading to faster hydration in the following order: HPMC K type > HPMC E type [24].

Figure 3b shows that the drug release increased with a decreasing HPMC viscosity in the following order: K15M ≈ K4M < K100LV < E15LV. This indicates that the drug release from these tablets was influenced by the viscosity of the hydrated gel layer. Higher viscosity HPMC formed a more tortuous and resistant barrier, slowing drug release. Similar results were observed with ciprofloxacin HCl tablets [25], where higher HPMC K15M viscosities prolonged the floating time and delayed the drug release rate. This suggests that high-viscosity HPMC forms highly viscous gels around the floating matrix tablet.

**Figure 4:**
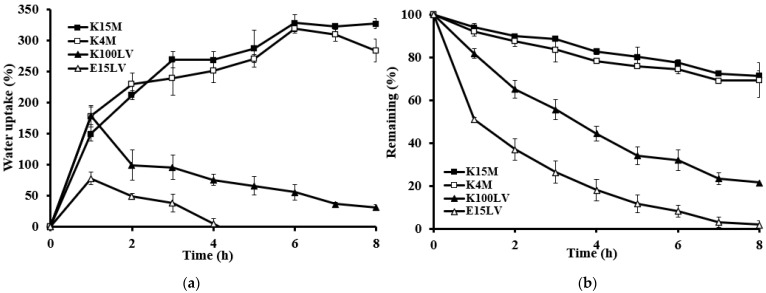
Percentage water uptake (**a**) and percentage erosion (**b**) of different HPMC viscosity grades of the floating matrix tablets (30% *w*/*w* menthol) in 0.1 N HCl.

These results were confirmed by swelling (i.e., water uptake) and erosion studies (Figure 4). Swelling behavior indicates the rate at which the tablet absorbs the medium and swells, while the remaining mass, measured by percentage weight decrease, reflects the erosion of the gel-forming polymer. The rate of water uptake was influenced by HPMC viscosity, as follows: high viscosity resulted in low erosion, whereas low viscosity resulted in high erosion. Thus, high-viscosity HPMC produced a stronger gel surrounding the tablet.

The type of HPMC used significantly influenced the floating force and drug release profiles of the tablets. Higher viscosity HPMC (e.g., K15M and K4M) formed a more robust gel layer, which enhanced the floating force and extended the buoyancy duration. Conversely, lower viscosity HPMC (e.g., E15LV and K100LV) resulted in a less stable floating force and a faster drug release due to a less resistant gel barrier.

#### 2.3.2. Effect of the Type of Sublimation Substances

Tablets with different types of sublimation substances were prepared and evaluated for their floating properties and drug release characteristics. As shown in Table 3, tablets containing 10% *w*/*w* camphor, borneol, and menthol floated immediately upon contact with the medium, while those with ammonium carbonate required 30% *w*/*w* to achieve immediate buoyancy. Tablets with 10% *w*/*w* ammonium carbonate had long float times of approximately 54 min, whereas those with 20% *w*/*w* floated within about 1 min, which was still significantly longer than the immediate float times observed for the camphor, borneol, and menthol tablets at 10% *w*/*w*.

**Figure 5:**
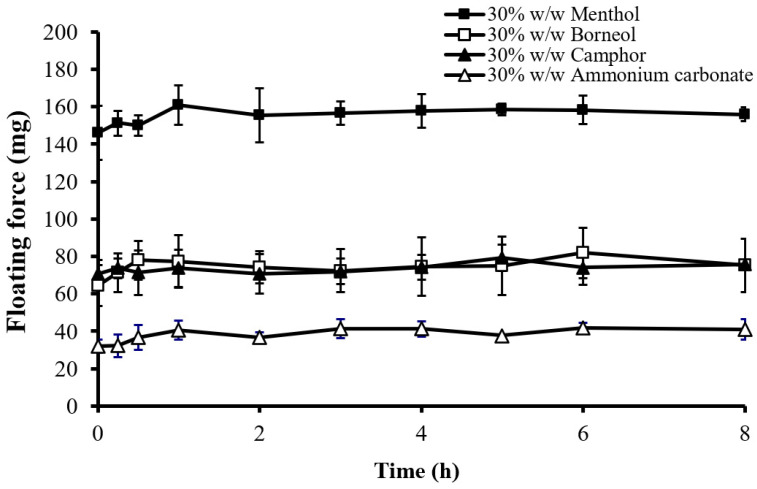
Effect of the type of sublimation substance on the floating force of the floating matrix tablets (HPMC K15M) in 0.1 N HCl.

Figure 5 shows the floating force of the tablets with various sublimation substances. Tablets with menthol exhibited the highest floating force, correlating with their lowest density and highest porosity (Table 1). This indicates that the type of sublimation substance significantly affects the floating force due to its influence on tablet density and porosity. The floating force was the highest for menthol, followed by camphor and borneol equally, and the lowest for ammonium carbonate. A higher floating force increases the probability of the tablet remaining afloat, which is crucial for extending gastric retention times [26].

**Figure 6:**
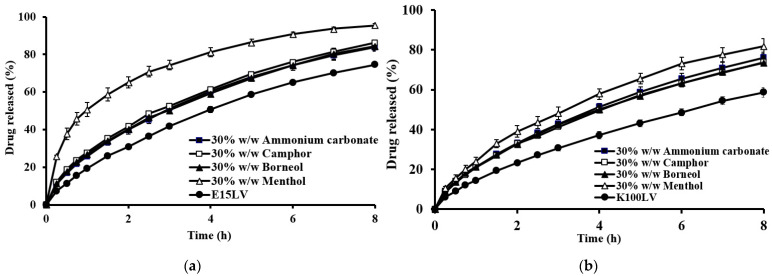
Effect of the types of sublimation substances on the theophylline released from floating matrix tablets in 0.1 N HCl: (**a**) E15LV; (**b**) K100LV.

**Figure 7:**
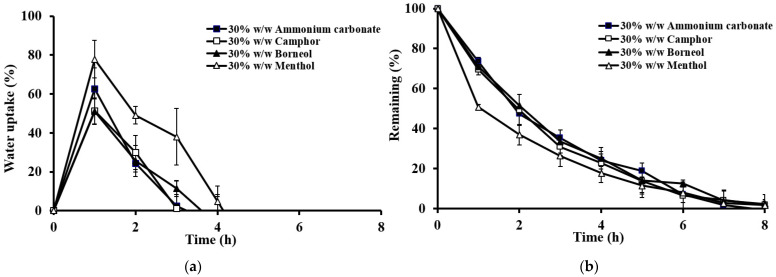
Percentage water uptake (**a**) and percentage erosion (**b**) for the floating matrix tablets (HPMC E15LV) with different types of sublimation substances in 0.1 N HCl.

The impact of the sublimation substance on drug release is shown in Figure 6. The tablets prepared with sublimation substances exhibited a noticeable increase in drug release compared to nonsublimation tablets. The type of sublimation substance significantly affected the theophylline release. For tablets with the lowest viscosity-grade HPMC (E15LV), those prepared with menthol released the drug significantly faster because of the higher porosity and lower hardness, as shown in Figure 6a. This result aligns with water uptake and erosion studies (Figure 7), where menthol tablets exhibited higher water uptake and erosion. The porous structure of the menthol tablets facilitated faster penetration of the dissolution medium, resulting in faster erosion and drug release. This concurs with previous findings that high porosity tablets (sponge-like structure) show greater dissolution medium penetration [27].

However, the increased drug release was less pronounced for tablets with HPMC K100LV (Figure 6b). For tablets using HPMC K4M and K15M, the type of sublimation substance had no significant effect on drug release. This is attributed to the strong gelatinous barrier formed by high-viscosity HPMC, which retards water penetration and drug dissolution.

#### 2.3.3. Effect of the Amount of Sublimation Substances

**Figure 8:**
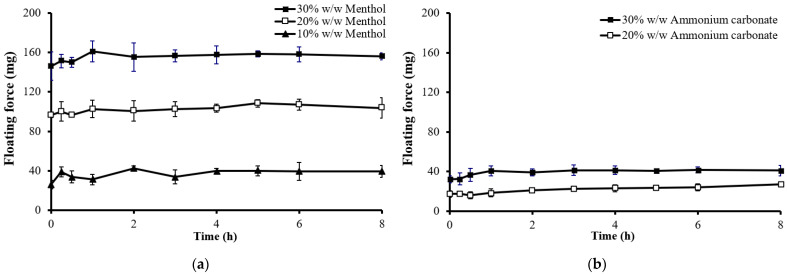
Effect of the amounts of sublimation substances on the floating force of floating matrix tablets (HPMC K15M) in 0.1 N HCl: (**a**) menthol; (**b**) ammonium carbonate.

The amount of sublimation substance is another crucial variable influencing the floating properties and drug release of matrix tablets. All formulations without sublimation substances failed to float. As the level of sublimation substance increased, the floating force increased, as illustrated in Figure 8. The required amount of sublimation substance varied depending on the type used. Tablets with camphor, borneol, and menthol floated with as little as 10% *w*/*w* sublimation substance, whereas more than 20% *w*/*w* ammonium carbonate was necessary to achieve immediate buoyancy (Table 2). Tablets with higher levels of sublimation substances exhibited greater porosity and lower density due to the sublimation process. Hence, optimizing the amount of sublimation substance is essential to ensure prolonged floatation with a consistent floating force.

**Figure 9:**
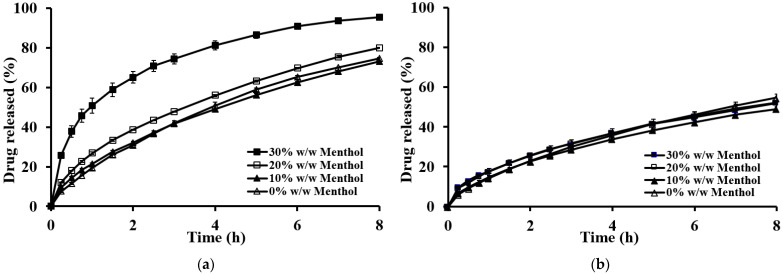
Effect of the amounts of sublimation substance (menthol) on theophylline released from floating matrix tablets in 0.1 N HCl: (**a**) E15LV; (**b**) K15M.

The impact of the amount of sublimation substance on drug release is shown in Figure 9. Drug release increased with higher amounts of sublimation substance, particularly for tablets with low-viscosity-grade HPMC (Figure 9a). This enhanced drug release is attributed to the water uptake and erosion rates of the tablets. A higher ratio of sublimation substance resulted in a more porous structure and lower polymer content, reducing tortuosity [28]. Consequently, the dissolution medium penetrated the tablet more rapidly, leading to faster erosion and drug release. However, for high-viscosity-grade HPMC (Figure 9b), the amount of sublimation substance had no significant effect on drug release. This was due to the ability of high-viscosity HPMC to form a strong gel barrier upon contact with acidic media, impeding water penetration and, thus, controlling drug release.

The quantity of sublimation substance used was directly correlated with the floating force and drug release rate. Higher amounts of sublimation substance increased the tablet porosity, reduced density, and enhanced both the floating force and drug release rate. However, excessive amounts could compromise the tablet’s mechanical strength, leading to lower hardness and higher friability. Optimizing the amount of sublimation substance is therefore crucial to balancing buoyancy with drug release stability.

### 2.4. Drug Release Kinetics

To study the release mechanism, various mathematical equations, including zero-order kinetics, first-order kinetics, Higuchi, and Korsmeyer–Peppas equations, were applied to the in vitro drug release data. The correlation coefficients are presented in Table 4. The drug release data for the tablets prepared using the sublimation technique showed a good fit with both the Higuchi equation and the Korsmeyer–Peppas equation.

The Higuchi model is applicable when the drug release is primarily governed by diffusion through water-filled pores in the matrix [29,30]. The good fit to the Korsmeyer–Peppas equation indicates that the release exponent ‘n’ values, determined from various formulations, ranged from 0.44 to 0.69, and the k value ranged from 0.140 to 0.497 (Table 4). The release exponents in the Korsmeyer–Peppas model suggested that theophylline was released according to anomalous (non-Fickian) diffusion-controlled release, which is a combined effect of diffusion and erosion mechanisms [31]. Additionally, the values of ‘n’ and k were found to increase with the amount of sublimation substance, indicating a direct relationship between the sublimation substance quantity and the drug release kinetics. This suggests that higher levels of sublimation substances enhance the porous structure of the tablets, facilitating a more pronounced diffusion and erosion process during drug release.

## 3. Conclusions

To address the issue of unpredictable floating times due to the variable pHs in gastric fluids, noneffervescent floating drug delivery systems were successfully developed using a novel technique based on sublimation substances. These systems demonstrated immediate floatation upon contact with the medium. The highly porous tablets, prepared by the evaporation of sublimation substances, exhibited reduced densities (<1 g/cm^3^). The gel-forming polymer swelled to form a viscous gel layer, ensuring prolonged floatation (over 8 h) and controlled drug release.

Tablets containing higher viscosity HPMC showed higher floating force, slower drug release, greater swelling, and slower erosion. Tablets with ammonium carbonate as the sublimation substance exhibited high hardness and low friability, while menthol-containing tablets displayed high porosity, high floating force, and faster drug release. Increasing the amount of sublimation substance enhanced porosity, reduced density, and increased both the floating force and drug release. However, high amounts of sublimation substance also resulted in a lower hardness and higher friability. The drug release data for the tablets prepared by the sublimation technique fit well with both the Higuchi and Korsmeyer–Peppas equations, indicating that the drug was released through an anomalous (non-Fickian) diffusion-controlled mechanism, which is a combination of diffusion and erosion.

In conclusion, the sublimation technique is a promising approach for developing gastroretentive drug delivery systems. This method is cost-effective and does not require special production equipment, making it a viable option for enhancing drug delivery in the stomach.

## 4. Materials and Methods

### 4.1. Materials

Anhydrous theophylline was purchased from Lianyungang Foreign Trade Corp. (Lianyungang, China). Hydroxypropyl methylcellulose (HPMC) (Methocel^®^ E15LV, K100LV, K4M, and K15M; Dow Chemical, Midland, MI, USA) served as the gel-forming polymer for the tablets. The viscosities of 2% aqueous solutions of E15LV, K100LV, K4M, and K15M were 15, 100, 4000, and 15,000 cps, respectively (at 20 °C). Sublimation substances included ammonium carbonate (Loba Chemie PVT, Mumbai, India), camphor and borneol (Suzhou Synthetic Chemical Co., Ltd., Suzhou, China), and menthol (Shanghai Xinjia Perfume Co., Ltd., Shanghai, China). Colloidal silicon dioxide (Aerosil^®^ 200, Degussa AG, Hanau, Germany) was used as a glidant, and magnesium stearate (Peter Greven Nederland C.V., Venlo, The Netherlands) as a lubricant. All other chemicals were of analytical or pharmaceutical grade.

### 4.2. Preparation of the Floating Matrix Tablets

The tablets were prepared by the direct compression method. The tablet components included the drug (anhydrous theophylline), filler (spray-dried lactose monohydrate, microcrystalline cellulose, or HPMC), and sublimation substances (ammonium carbonate, camphor, borneol, or menthol), as described in Table 4. All excipients for the floating matrix tablets were passed through a 60-mesh sieve before being accurately weighed and mixed using the geometric dilution technique for 15 min. Magnesium stearate (0.5% *w*/*w*) and Aerosil^®^ 200 (0.5% *w*/*w*) were then added to the mixture, which was further mixed for 3 min. The powder mixture was compressed into biconvex tablets (diameter: 9.53 mm; hardness: 9–10 kg; and average tablet weight: 300 mg) using a hydraulic press tableting machine (Specac Inc., Cranston, RI, USA). The matrix tablets were incubated in an oven at 70 °C for 72 h to eliminate sublimation substances. A consistent compression force of 1 ton was applied for 5 s across all formulations. The tablets were stored over silica gel at room temperature until required for further investigation.

### 4.3. Evaluation of the Floating Matrix Tablets

#### 4.3.1. Evaluation of the Floating Matrix Tablet Properties

The floating matrix tablets were assessed for several physical properties, including uniformity of weight, thickness, diameter, hardness, and friability. The thickness and diameter of the tablets were measured with a thickness tester (Micrometer Caliper, Model 7301 Series 7, Mitutoyo, Kawasaki, Japan). The hardness was determined with a texture analyzer (TA.XT.plus, Stable Micro Systems, Godalming, UK). The friability was assessed using a friability test apparatus (Model 45-2200, Vankel, Cary, NC, USA).

The apparent density (*ρ_a_*) of the tablets (g/cm^3^) was calculated based on tablet height, diameter, and mass using the following equation:

*ρ_a_* = *W*/[*π*(*d*/2)^2^
*h*], (1)

where *W* is the mass of a tablet, *π* is the circular constant, *d* is the tablet diameter, and *h* is the tablet height.

The theophylline content in the tablets was determined by methanol extraction. Accurately weighed and ground tablets were transferred to a volumetric flask; after that, methanol was added and stirred for 2 h to ensure a complete extraction. The mixture was passed through filter paper, diluted with the appropriate volume of methanol, and assayed spectrophotometrically at 270 nm using a spectrophotometer (Model UV2450, Shimadzu, Kyoto, Japan). Each formulation was assessed in triplicate.

#### 4.3.2. Porosity Determination of the Floating Matrix Tablets

The porosity (*ε*) of the tablets was calculated using following equation:

*Porosity* (*ε*) = (1 − *ρ_a_*/*ρ_t_*) × 100, (2)

The true density (*ρ_t_*) of the tablets was determined using a helium displacement device (Pycnometer 1305, Micromeritics, Norcross, GA, USA). The true volume of the tablets was calculated by measuring the volume of helium displaced by the tablets. True density was then calculated by dividing the tablet weight by the true volume. Each formulation was analyzed in triplicate. The apparent densities (*ρ_a_*) of the floating matrix tablet were calculated using Equation (1) (as described earlier).

#### 4.3.3. Scanning Electron Microscopy (SEM)

The dried tablets were mounted on metal stubs and coated with 30 nm of gold under vacuum. The surface and cross-section morphology of the tablets were then observed using scanning electron microscopy (Maxxim-2000, CamScan Analytical, London, UK).

#### 4.3.4. Floating Properties

The floating properties of the tablets were evaluated using the USP dissolution apparatus II containing 900 mL of 0.1 N HCl as the medium, maintained at 37 °C. The following two key parameters were measured: (1) Time to Float: The time required for the tablet to rise from the bottom to the top of the vessel; (2) Floating Duration (Floating Time): The total period during which the tablet remained afloat after being placed in the dissolution medium until it eventually sank.

#### 4.3.5. Resultant Weight

To monitor in vitro the total vertical force (*F*) working on an immersed tablet, resultant-weight measurements were performed using a previously described apparatus and methodology [27,32,33]. This force quantifies and characterizes the floating behavior of the tablet. The total vertical force (*F*) is the vectorial sum of the gravity force (*F_grav_*) and buoyancy force (*F_buoy_*) acting on the tablet, determined by the following equation:

*F* = *F_buoy_* − *F_grav_* *F* = *d_f_gV* − *d_s_gV* *F* = (*d_f_* − *d_s_*) *gV*, (3)

where *F* is the total vertical force, *g* is the acceleration of gravity, *d_f_* is the density of the fluid, *d_s_* is the density of the tablet, and *V* is the volume of the tablet.

Floating force experiments were conducted by placing the tablets in a beaker containing 300 mL of 0.1 N HCl at 37 °C ± 0.5 °C. A probe maintained the tablet submerged at a constant depth of 20 mm throughout the measurement. The floating force was determined as the total vertical force recorded by the texture analyzer over time.

#### 4.3.6. Drug Release Studies

Drug release studies were conducted using 900 mL of 0.1 N HCl as the dissolution medium in a USP dissolution apparatus II (UDT 804, Logan Instruments Corp., Somerset, NJ, USA) maintained at 37 ± 0.5 °C and 50 rpm. At predetermined time points, 5 mL samples were withdrawn and replaced with fresh medium to maintain a constant volume. The amount of theophylline release was determined using a UV/visible spectrophotometer (Model UV2450, Shimadzu, Kyoto, Japan) at a wavelength of 270 nm, using a 1.0 cm quartz cell. Each formulation was tested for a minimum of three replicates.

#### 4.3.7. Swelling or Water Uptake Studies

The swelling of the tablets was assessed by their ability to absorb water and swell. The tablets were accurately weighed (*W*_0_), placed in closed plastic containers with the mesh underneath, and rotated at 150 rpm in an Environmental Shaker-Incubator (Model ES-20, Biosan, Riga, Latvia) containing 0.1 N HCl as the medium, maintained at 37 °C ± 0.5 °C. At predetermined time intervals, the tablets with the pre-weighed mesh were removed, lightly blotted with tissue paper to eliminate excess liquid, and re-weighed (*W*_1_). The experiment was performed in triplicate for each time point, using fresh samples each time. The percentage increase in weight due to water uptake was calculated using the following equation [34]:

% *water uptake* = (*W*_1_ − *W*_0_)/*W*_0_ × 100, (4)

#### 4.3.8. Erosion Studies

Following the swelling studies, wet samples were dried in an oven at 80 °C for 24 h, then allowed to cool in a desiccator until a constant weight was achieved (final dry weight, *W*_2_). The experiment was performed in triplicate for each time point. The percentage of tablet erosion and the remaining mass were calculated using the following equations [34]:

% *erosion* = [(*W*_0_ − *W*_2_)/*W*_0_] × 100, (5)

% *remaining* = 1 − % *erosion*, (6)

#### 4.3.9. Analysis of Drug Release Data

The mechanism of drug release from the tablets during dissolution tests was determined using a zero-order kinetics equation, first-order kinetics equation, Higuchi equation, and Korsmeyer–Peppas equation. The zero-order kinetics equation (Equation (7)) describes the system in which the drug release rate is independent of its concentration.

*C* = *k*_0_t, (7)

where *k*_0_ is the zero-order rate constant expressed in units of concentration/time, and *t* is the time.

The first-order kinetics Equation (8) describes the drug release profile from the system, where the release rate is concentration-dependent.

*Log C* = *Log C*_0_ − *kt*/2.303, (8)

where *C*_0_ is the initial concentration of drug, and *k* is the first-order constant.

The Higuchi equation describes the release of drugs from insoluble matrix as a square root of time dependent process based on Fickian diffusion (Equation (9)).

*Q* = *kt*^1/2^, (9)

where *k* is the constant reflecting the design variables of the system.

The Korsmeyer–Peppas equation describes the drug release from a polymeric system (Equation (10)).

*M_t_*/*M*_∞_ = *kt^n^*, (10)

where *M_t_*/*M*_∞_ is the fraction of drug released at time *t*, *k* is a constant incorporating geometric and structural characteristics, and *n* is an exponent that indicates the drug release mechanism.

When determining the *n* exponent, only the portions of the release profile where (*M_t_*/*M*_∞_) ≤ 0.6 were used to provide the accurate values [31]. The exponent *n* takes a limiting value of 0.45, it is a case of diffusion-controlled drug release (Fickian release). Case II transport or relaxation-controlled delivery; the exponent *n* is 0.89 for release from the cylinder. Values of *n* between 0.45 and 0.89 can be regarded as indicating non-Fickian release or anomalous transport. The non-Fickian kinetics are regarded as coupled diffusion and polymer relaxation. In addition, Super Case II kinetics are considered when the values of *n* > 0.89 for the release from the cylinder are observed.

## Figures and Tables

**Table 1:** Physical characteristics of the floating matrix tablets after drying by incubation at 70 °C for 72 h.

Formulation	Wt. Variation (mg)	Thickness (mm)	Density (mg/mm^3^)	Porosity (%)	Hardness (kg)	Friability (%)
HPMC E15LV	302.62 ± 3.39	3.95 ± 0.03	1.14 ± 0.01	15.84 ± 1.05	14.04 ± 0.28	−0.25
10% *w*/*w* Ammonium carbonate	271.48 ± 2.77	3.81 ± 0.04	1.05 ± 0.01	26.96 ± 1.72	8.37 ± 0.31	−0.11
20% *w*/*w* Ammonium carbonate	240.23 ± 4.45	3.84 ± 0.05	0.94 ± 0.02	31.23 ± 1.56	5.19 ± 0.27	−0.12
30% *w*/*w* Ammonium carbonate	214.09 ± 5.37	3.70 ± 0.12	0.82 ± 0.03	41.32 ± 2.75	1.85 ± 0.35	−0.32
10% *w*/*w* Camphor	272.38 ± 4.46	4.09 ± 0.06	0.91 ± 0.01	31.22 ± 2.33	4.43 ± 0.56	0.07
20% *w*/*w* Camphor	238.48 ± 6.22	4.07 ± 0.15	0.80 ± 0.02	37.35 ± 1.16	3.34 ± 0.33	0.65
30% *w*/*w* Camphor	212.33 ± 5.02	4.01 ± 0.05	0.73 ± 0.06	47.72 ± 1.56	2.31 ± 0.18	1.83
10% *w*/*w* Borneol	276.32 ± 1.91	4.04 ± 0.03	0.91 ± 0.01	30.71 ± 1.89	5.33 ± 0.31	−0.34
20% *w*/*w* Borneol	245.38 ± 4.24	4.57 ± 0.13	0.83 ± 0.03	37.69 ± 1.25	2.99 ± 0.15	−0.04
30% *w*/*w* Borneol	208.07 ± 4.72	4.00 ± 0.05	0.72 ± 0.03	45.24 ± 1.85	2.03 ± 0.24	0.30
10% *w*/*w* Menthol	275.58 ± 5.02	4.70 ± 0.09	0.81 ± 0.02	35.19 ± 0.35	8.48 ± 0.53	−0.04
20% *w*/*w* Menthol	244.71 ± 4.70	5.09 ± 0.20	0.66 ± 0.02	45.69 ± 1.94	4.91 ± 0.60	1.48
30% *w*/*w* Menthol	209.26 ± 3.89	6.25 ± 0.07	0.46 ± 0.04	58.83 ± 1.85	1.71 ± 0.19	32.54
HPMC K100LV	304.92 ± 2.46	3.82 ± 0.06	1.13 ± 0.01	14.43 ± 0.80	38.95 ± 1.47	−0.16
30% *w*/*w* Ammonium carbonate	213.19 ± 5.14	3.56 ± 0.08	0.91 ± 0.04	29.62 ± 0.80	15.73 ± 0.77	0.07
30% *w*/*w* Camphor	216.03 ± 4.32	4.12 ± 0.06	0.71 ± 0.03	42.55 ± 0.96	6.32 ± 0.37	1.21
30% *w*/*w* Borneol	212.35 ± 5.66	4.06 ± 0.03	0.72 ± 0.02	41.47 ± 1.66	7.16 ± 0.56	0.47
30% *w*/*w* Menthol	209.86 ± 4.30	4.90 ± 0.10	0.58 ± 0.02	53.15 ± 2.32	1.10 ± 0.21	6.41
HPMC K4M	307.82 ± 3.01	3.79 ± 0.04	1.13 ± 0.01	14.34 ± 0.11	33.91 ± 0.86	0.07
30% *w*/*w* Ammonium carbonate	220.60 ± 7.37	3.49 ± 0.05	0.87 ± 0.02	30.11 ± 2.71	7.88 ± 0.43	0.11
30% *w*/*w* Camphor	214.32 ± 5.29	4.02 ± 0.08	0.75 ± 0.13	40.10 ± 2.62	5.36 ± 0.19	0.07
30% *w*/*w* Borneol	210.91 ± 4.11	3.99 ± 0.04	0.74 ± 0.02	39.93 ± 1.61	5.34 ± 0.76	0.04
30% *w*/*w* Menthol	213.53 ± 4.06	4.60 ± 0.06	0.62 ± 0.02	51.47 ± 2.30	0.76 ± 0.06	0.55
HPMC K15M	304.10 ± 0.58	3.70 ± 0.04	1.15 ± 0.01	15.08 ± 1.03	30.57 ± 0.62	−0.09
10% *w*/*w* Ammonium carbonate	268.29 ± 2.54	3.63 ± 0.05	1.06 ± 0.02	21.74 ± 1.99	22.25 ± 2.13	−0.13
20% *w*/*w* Ammonium carbonate	243.59 ± 5.64	3.65 ± 0.03	0.96 ± 0.02	30.24 ± 2.25	14.14 ± 1.03	0.93
30% *w*/*w* Ammonium carbonate	215.04 ± 5.46	3.55 ± 0.05	0.84 ± 0.06	37.13 ± 0.97	8.27 ± 0.13	4.68
10% *w*/*w* Camphor	271.35 ± 2.93	4.06 ± 0.03	0.94 ± 0.02	30.38 ± 2.36	8.81 ± 0.90	−0.50
20% *w*/*w* Camphor	241.58 ± 3.36	4.07 ± 0.04	0.80 ± 0.06	36.23 ± 2.01	5.98 ± 0.43	3.26
30% *w*/*w* Camphor	216.18 ± 10.98	3.96 ± 0.03	0.74 ± 0.04	47.35 ± 1.22	4.35 ± 0.45	5.94
10% *w*/*w* Borneol	269.18 ± 1.06	4.00 ± 0.06	0.94 ± 0.01	28.06 ± 2.30	12.02 ± 0.73	0.81
20% *w*/*w* Borneol	242.97 ± 6.68	4.01 ± 0.05	0.86 ± 0.03	36.34 ± 2.11	8.37 ± 1.08	3.60
30% *w*/*w* Borneol	212.01 ± 5.31	4.07 ± 0.06	0.70 ± 0.02	44.04 ± 2.13	3.52 ± 0.59	4.65
10% *w*/*w* Menthol	275.24 ± 2.87	4.22 ± 0.09	0.91 ± 0.01	35.31 ± 0.69	6.27 ± 0.45	0.30
20% *w*/*w* Menthol	243.95 ± 3.87	4.84 ± 0.22	0.68 ± 0.04	49.24 ± 2.42	1.56 ± 0.20	0.12
30% *w*/*w* Menthol	213.31 ± 5.37	5.13 ± 0.07	0.56 ± 0.01	57.96 ± 3.25	0.60 ± 0.05	3.44

**Table 2:** Floating properties of the floating matrix tablets.

Formulation	Time to Float (min ± SD)	Floating Time (h)
HPMC E15LV	NF	-
10% *w*/*w* Ammonium carbonate	54.40 ± 4.77	>8
20% *w*/*w* Ammonium carbonate	1.16 ± 0.41	>8
30% *w*/*w* Ammonium carbonate	IF	>8
10% *w*/*w* Camphor	IF	>8
20% *w*/*w* Camphor	IF	>8
30% *w*/*w* Camphor	IF	>8
10% *w*/*w* Borneol	IF	>8
20% *w*/*w* Borneol	IF	>8
30% *w*/*w* Borneol	IF	>8
10% *w*/*w* Menthol	IF	>8
20% *w*/*w* Menthol	IF	>8
30% *w*/*w* Menthol	IF	>8
HPMC K100LV	NF	-
30% *w*/*w* Ammonium carbonate	IF	>8
30% *w*/*w* Camphor	IF	>8
30% *w*/*w* Borneol	IF	>8
30% *w*/*w* Menthol	IF	>8
HPMC K4M	NF	-
30% *w*/*w* Ammonium carbonate	IF	>8
30% *w*/*w* Camphor	IF	>8
30% *w*/*w* Borneol	IF	>8
30% *w*/*w* Menthol	IF	>8
HPMC K15M	NF	-
10% *w*/*w* Ammonium carbonate	57.97 ± 3.26	>8
20% *w*/*w* Ammonium carbonate	1.62 ± 0.47	>8
30% *w*/*w* Ammonium carbonate	IF	>8
10% *w*/*w* Camphor	IF	>8
20% *w*/*w* Camphor	IF	>8
30% *w*/*w* Camphor	IF	>8
10% *w*/*w* Borneol	IF	>8
20% *w*/*w* Borneol	IF	>8
30% *w*/*w* Borneol	IF	>8
10% *w*/*w* Menthol	IF	>8
20% *w*/*w* Menthol	IF	>8
30% *w*/*w* Menthol	IF	>8

NF = non-floating; IF = initially floated.

**Table 3:** Mathematic modeling and drug release kinetics of theophylline from the floating matrix tablets.

Formulation	Correlation Coefficient, *r*^2^				Kinetics Constant, k (Korsmeyer– Peppas Model) (h^−n^)	Diffusional Exponent, n (Korsmeyer– Peppas Model)	Order of Release (Korsmeyer– Peppas Model)
	Zero Order	First Order	Higuchi Order	Korsmeyer–Peppas Model			
HPMC E15LV	0.9631	0.6015	0.9889	0.9992	0.1929	0.6961	Anomalous (non-Fickian) diffusion
10% *w*/*w* Ammonium carbonate	0.9417	0.5214	0.9973	0.9997	0.2590	0.5990	Anomalous (non-Fickian) diffusion
20% *w*/*w* Ammonium carbonate	0.9538	0.5529	0.9947	0.9991	0.2332	0.6153	Anomalous (non-Fickian) diffusion
30% *w*/*w* Ammonium carbonate	0.9398	0.5233	0.9966	0.9998	0.2607	0.6103	Anomalous (non-Fickian) diffusion
10% *w*/*w* Camphor	0.9588	0.5619	0.9938	0.9997	0.2306	0.6489	Anomalous (non-Fickian) diffusion
20% *w*/*w* Camphor	0.9609	0.5573	0.9938	0.9993	0.2314	0.6126	Anomalous (non-Fickian) diffusion
30% *w*/*w* Camphor	0.9352	0.5028	0.9982	0.9992	0.2796	0.5834	Anomalous (non-Fickian) diffusion
10% *w*/*w* Borneol	0.9616	0.5715	0.9934	0.9997	0.2103	0.6379	Anomalous (non-Fickian) diffusion
20% *w*/*w* Borneol	0.9644	0.5720	0.9927	0.9995	0.2134	0.6345	Anomalous (non-Fickian) diffusion
30% *w*/*w* Borneol	0.9416	0.5152	0.9979	0.9988	0.2656	0.5960	Anomalous (non-Fickian) diffusion
10% *w*/*w* Menthol	0.9583	0.5532	0.9955	0.9998	0.2163	0.5904	Anomalous (non-Fickian) diffusion
20% *w*/*w* Menthol	0.9401	0.5028	0.9992	0.9994	0.2628	0.5508	Anomalous (non-Fickian) diffusion
30% *w*/*w* Menthol	0.7787	0.3234	0.9511	0.9871	0.4972	0.4435	Fickian diffusion
HPMC K100LV	0.9856	0.6387	0.9950	0.9998	0.1452	0.6765	Anomalous (non-Fickian) diffusion
30% *w*/*w* Ammonium carbonate	0.9600	0.5752	0.9932	0.9997	0.2087	0.6535	Anomalous (non-Fickian) diffusion
30% *w*/*w* Camphor	0.9599	0.5638	0.9942	0.9992	0.2121	0.6097	Anomalous (non-Fickian) diffusion
30% *w*/*w* Borneol	0.9562	0.5613	0.9952	0.9994	0.2116	0.6214	Anomalous (non-Fickian) diffusion
30% *w*/*w* Menthol	0.9460	0.5392	0.9954	0.9982	0.2455	0.6201	Anomalous (non-Fickian) diffusion
HPMC K4M	0.9675	0.9631	0.9910	0.9996	0.1409	0.6476	Anomalous (non-Fickian) diffusion
30% *w*/*w* Ammonium carbonate	0.9479	0.5658	0.9969	0.9992	0.1800	0.5914	Anomalous (non-Fickian) diffusion
30% *w*/*w* Camphor	0.9558	0.5969	0.9948	0.9985	0.1528	0.6121	Anomalous (non-Fickian) diffusion
30% *w*/*w* Borneol	0.9427	0.5581	0.9985	0.9988	0.1714	0.5731	Anomalous (non-Fickian) diffusion
30% *w*/*w* Menthol	0.9523	0.5479	0.9970	0.9992	0.1800	0.5349	Anomalous (non-Fickian) diffusion
HPMC K15M	0.9683	0.6253	0.9906	0.9996	0.1448	0.6474	Anomalous (non-Fickian) diffusion
10% *w*/*w* Ammonium carbonate	0.9568	0.5944	0.9935	0.9960	0.1731	0.6452	Anomalous (non-Fickian) diffusion
20% *w*/*w* Ammonium carbonate	0.9588	0.5739	0.9946	0.9994	0.1941	0.6158	Anomalous (non-Fickian) diffusion
30% *w*/*w* Ammonium carbonate	0.9526	0.5822	0.9955	0.9988	0.1716	0.6087	Anomalous (non-Fickian) diffusion
10% *w*/*w* Camphor	0.9563	0.5820	0.9944	0.9991	0.1807	0.6171	Anomalous (non-Fickian) diffusion
20% *w*/*w* Camphor	0.9495	0.5601	0.9981	0.9997	0.1709	0.5515	Anomalous (non-Fickian) diffusion
30% *w*/*w* Camphor	0.9621	0.5739	0.9938	0.9986	0.1682	0.5670	Anomalous (non-Fickian) diffusion
10% *w*/*w* Borneol	0.9619	0.5958	0.9925	0.9991	0.1716	0.6344	Anomalous (non-Fickian) diffusion
20% *w*/*w* Borneol	0.9460	0.5689	0.9979	0.9990	0.1649	0.5726	Anomalous (non-Fickian) diffusion
30% *w*/*w* Borneol	0.9657	0.5860	0.9921	0.9988	0.1670	0.5902	Anomalous (non-Fickian) diffusion
10% *w*/*w* Menthol	0.9511	0.5879	0.9967	0.9993	0.1492	0.5885	Anomalous (non-Fickian) diffusion
20% *w*/*w* Menthol	0.9379	0.5345	0.9993	0.9998	0.1748	0.5292	Anomalous (non-Fickian) diffusion
30% *w*/*w* Menthol	0.9339	0.5215	0.9995	0.9989	0.1803	0.5040	Anomalous (non-Fickian) diffusion

**Table 4:** Composition of the floating matrix tablet formulations.

Component (mg)	Formulation																	
	1	2	3	4	5	6	7	8	9	10	11	12	13	14	15	16	17	18
Theophylline	20	20	20	20	20	20	20	20	20	20	20	20	20	20	20	20	20	20
Methocel^®^ E15LV	280	190	220	250	190	220	250	190	220	250	190	220	250	-	-	-	-	-
Methocel^®^ K100LV	-	-	-	-	-	-	-	-	-	-	-	-	-	280	190	190	190	190
Methocel^®^ K4M	-	-	-	-	-	-	-	-	-	-	-	-	-	-	-	-	-	-
Methocel^®^ K15M	-	-	-	-	-	-	-	-	-	-	-	-	-	-	-	-	-	-
Ammonium carbonate	-	90	60	30	-	-	-	-	-	-	-	-	-	-	90	-	-	-
Camphor	-	-	-	-	90	60	30	-	-	-	-	-	-	-	-	90	-	-
Borneol	-	-	-	-	-	-	-	90	60	30	-	-	-	-	-	-	90	-
Menthol	-	-	-	-	-	-	-	-	-	-	90	60	30	-	-	-	-	90
Magnesium stearate	0.5%	0.5%	0.5%	0.5%	0.5%	0.5%	0.5%	0.5%	0.5%	0.5%	0.5%	0.5%	0.5%	0.5%	0.5%	0.5%	0.5%	0.5%
Aerosil^®^ 200	0.5%	0.5%	0.5%	0.5%	0.5%	0.5%	0.5%	0.5%	0.5%	0.5%	0.5%	0.5%	0.5%	0.5%	0.5%	0.5%	0.5%	0.5%
**Component (mg)**	**Formulation**																	
	**19**	**20**	**21**	**22**	**23**	**24**	**25**	**26**	**27**	**28**	**29**	**30**	**31**	**32**	**33**	**34**	**35**	**36**
Theophylline	20	20	20	20	20	20	20	20	20	20	20	20	20	20	20	20	20	20
Methocel^®^ E15LV	-	-	-	-	-	-	-	-	-	-	-	-	-	-	-	-	-	-
Methocel^®^ K100LV	-	-	-	-	-	-	-	-	-	-	-	-	-	-	-	-	-	-
Methocel^®^ K4M	280	190	190	190	190	-	-	-	-	-	-	-	-	-	-	-	-	-
Methocel^®^ K15M	-	-	-	-	-	280	190	220	250	190	220	250	190	220	250	190	220	250
Ammonium carbonate	-	90	-	-	-	-	90	60	30	-	-	-	-	-	-	-	-	-
Camphor	-	-	90	-	-	-	-	-	-	90	60	30	-	-	-	-	-	-
Borneol	-	-	-	90	-	-	-	-	-	-	-	-	90	60	30	-	-	-
Menthol	-	-	-	-	90	-	-	-	-	-	-	-	-	-	-	90	60	30
Magnesium stearate	0.5%	0.5%	0.5%	0.5%	0.5%	0.5%	0.5%	0.5%	0.5%	0.5%	0.5%	0.5%	0.5%	0.5%	0.5%	0.5%	0.5%	0.5%
Aerosil^®^ 200	0.5%	0.5%	0.5%	0.5%	0.5%	0.5%	0.5%	0.5%	0.5%	0.5%	0.5%	0.5%	0.5%	0.5%	0.5%	0.5%	0.5%	0.5%

## Data Availability

The data that support the findings of this study are available from the corresponding author upon reasonable request. Data are stored in a secure institutional repository and are subject to access restrictions due to privacy concerns.
